# Denervation Atrophy of Skeletal Muscle is Not Influenced by Numb Levels in Mice

**DOI:** 10.7150/ijms.77603

**Published:** 2023-01-31

**Authors:** Abdurrahman Aslan, Lauren Harlow, Xin-Hua Liu, Rita De Gasperi, William A. Bauman, Marco Brotto, Christopher P Cardozo

**Affiliations:** 1 National Center for the Medical Consequences of Spinal Cord Injury, James J. Peters VA.; 2 Department of Medicine, Icahn School of Medicine at Mount Sinai.; 3 Department of Rehabilitation Medicine, Icahn School of Medicine at Mount Sinai.; 4 Mount Sinai Institute for Systems Biomedicine, Icahn School of Medicine at Mount Sinai.; 5 Department of Psychiatry and Friedman Brain Institute, Icahn School of Medicine at Mount Sinai.; 6 Bone-Muscle Research Center, College of Nursing & Health Innovation, The University of Texas at Arlington, Arlington, TX 76019.

**Keywords:** skeletal muscle, numb, denervation atrophy, nandrolone, aging, sarcopenia

## Abstract

Skeletal muscle undergoes rapid and extensive atrophy following nerve transection though the underlying mechanisms remain incompletely understood. We previously showed transiently elevated Notch 1 signaling in denervated skeletal muscle that was abrogated by administration of nandrolone (an anabolic steroid) combined with replacement doses of testosterone. Numb is an adaptor molecule present in myogenic precursors and skeletal muscle fibers that is vital for normal tissue repair after muscle injury and for skeletal muscle contractile function. It is unclear whether the increase in Notch signaling observed in denervated muscle contributes to denervation and whether expression of Numb in myofibers slows denervation atrophy. To address these questions, the degree of denervation atrophy, Notch signaling, and Numb expression was studied over time after denervation in C57B6J mice treated with nandrolone, nandrolone plus testosterone or vehicle. Nandrolone increased Numb expression and reduced Notch signaling. Neither nandrolone alone nor nandrolone plus testosterone changed the rate of denervation atrophy. We next compared rates of denervation atrophy between mice with conditional, tamoxifen-inducible knockout of Numb in myofibers and genetically identical mice treated with vehicle. Numb cKO had no effect on denervation atrophy in this model. Taken together, the data indicate that loss of Numb in myofibers does not alter the course of denervation atrophy and that upregulation of Numb and blunting of the denervation-atrophy induced activation of Notch do not change the course of denervation atrophy.

## Introduction

Skeletal muscle demonstrates marked and rapid atrophy upon nerve transection 1. Abundant evidence links increased destruction of sarcomeric proteins via the ubiquitin-proteasome system to denervation atrophy 2, 3. Several key signals have been identified as critical drivers of denervation atrophy. Studies from the Kandarian lab showed critical roles for activation of NF-kB 4. Studies with mice lacking the expression connexin (Cx) 43 and 45 in skeletal muscle fibers implicated de-novo sarcoplasmic integration of these membrane channels in denervation atrophy, reduction of resting membrane potential, activation of NF-kB and entry of sodium and calcium into denervate myofibers 5, 6. It was later shown that miniature end plate potentials stimulated by quantal release of acetylcholine from nerve terminals were sufficient to prevent appearance of Cx in the sarcolemma and thereby prevent denervation atrophy 7.

In Sprague Dawley rats, the anabolic steroid nandrolone (Nan) slowed denervation atrophy slightly though significantly when treatment was combined with testosterone and started at day 29 after nerve transection 8. Studies of the underlying mechanism found that, in rats, denervation atrophy was associated with a transient elevation in Notch1 signaling via increased nuclear levels of Notch1 intracellular domain (NICD) and upregulation of the Notch target gene Hey1 9. Treatment with Nan reduced the nuclear levels of Notch intracellular domain (NICD) and the expression of Hey1 to baseline values 9 and resulted in elevated levels of the adaptor protein Numb. Effects of Nan on changes in Notch1 expression and activation may depend on the model of muscle atrophy. For example, in one study of hind-limb unloading, Notch1 expression was decreased, and this change was prevented by 14 days pre-treatment with Nan 10.

Notch 1 is a member of the Notch family of cell-surface receptors. Signaling via Notch is activated by binding of specific ligands which trigger release of the NICD which is translocated to the nucleus where it regulates gene expression 11. Notch promotes proliferation of satellite cells and suppresses their myogenic differentiation 12, 13. More recent studies using genetically modified mice implicate Notch signaling in maintaining healthy myofibers 14. Paradoxically perhaps, Notch signaling has been implicated in muscle loss due to cancer cachexia 15; Notch signaling was elevated in atrophied muscles of mice bearing tumors while a Notch inhibitor reduced atrophy 15. Thus, Notch is required for skeletal muscle fiber homeostasis but excessive Notch signaling may stimulate muscle atrophy. It is unclear whether Notch signaling contributes to denervation-related atrophy.

Numb is an adaptor protein best known for its role in asymmetric division of progenitors in nervous system development 16. It has been implicated as having critical roles in many contexts that include development of the central nervous system and heart 16, 17, inhibition of sonic hedgehog signaling through the E3 ubiquitin ligase Itch 18, targeting Notch 1 and NICD for proteolytic destruction via the ubiquitin-proteasome pathway 16 and cancer biology 19, 20. Numb plays critical roles in suppressing Notch signaling in response to canonical Wnt signaling 21, a critical step in cell fate determination. Numb expression is upregulated by canonical Wnt signaling via recruitment of TCF-LEF family members to the Numb promoter 22. In skeletal muscle, decreased expression of Numb in satellite cells delays tissue repair and reduces body and muscle size, possibly through upregulation of myostatin 23. Numb is expressed at high levels in skeletal muscle fibers where it is required for optimal muscle contractility and sarcomeric structure. Numb level decreases in mouse gastrocnemius muscle with aging suggesting that aging related muscle weakness may be the result of reduced Numb expression 24. Whether Numb protects against skeletal muscle atrophy is unknown.

The objective of the current study was to better understand the possible role(s) of Numb in skeletal muscle fibers in denervation atrophy. Our hypothesis was that genetic ablation of Numb expression in skeletal muscle fibers would accelerate denervation atrophy and that, by abrogating denervation-induced increases in Notch signaling, upregulation of Numb by nandrolone would slow denervation atrophy. To test if loss of Numb in skeletal muscle fibers changed the rate of denervation atrophy, we used genetically modified mice in which Numb knockouts can be conditionally induced in myofibers by injection of tamoxifen. To understand whether upregulation of Numb and abrogation of denervation-induced increases in Notch signaling slowed denervation atrophy, we evaluated muscle atrophy, Notch signaling and Numb levels in vehicle or nandrolone-treated mice. These studies showed no influence of either nandrolone-induced increased Numb or genetically ablated Numb levels on rates of denervation atrophy in mice.

## Methods

### Animals

Fifteen-week male C57B6J (Stock Number 000664) mice, weighing 25 ± 3g were obtained from Jackson Laboratories, Harbor, ME. HSA-MCM/Numb^(f/f)^/NumbL^(f/f)^ mice which allow a conditional, inducible knockout of Numb and the closely related gene Numb-Like in skeletal muscle fibers have been previously described 24. The timeline of the experiments and number of animals used for this experiment is shown (Supplementary Fig. 4). All pups were genotyped prior to use in breeding or experiments 24. All studies with animal subjects were approved by the Institutional Animal Use and Care Committee of the James J Peters VA and were conducted in accordance with all applicable Federal rules and regulations (protocol CAR-11-51).

### Denervation

Denervation was accomplished by cutting the left sciatic nerve as described 8 with minor modifications. Briefly, animals were anesthetized by inhalation of isoflurane at 3.5% for induction and 2.5-3% for maintenance. Hair was removed over the left hip with a clipper and skin was cleaned with 70% ethanol and beta-iodine solution. Animals were placed on a drape over a warming pad heated with recirculating water at 37 °C. The sciatic nerve was visualized through a small skin incision just posterior to the head of the femur and aligned with the femur by blunt dissection. After excising a segment of the sciatic nerve just proximal to the trifurcation, wounds were closed in layers with sutures. All animals were administered 5 mg/kg Carprofen, Baytril, and Ringer's lactate before the surgery and for 3 days post-surgery. The contralateral leg was used as control as it is shown in the figure legends.

### Drug Administration

Alzet mini osmotic pumps filled with propylene glycol (Sigma, 25322-69-4) containing androgens were used at doses described under methods for individual experiments and in the figure legends or propylene glycol alone were used. Pumps were prepared and primed 24-48 hours before implantation following the manufacturer's protocol to insure immediate administration of the drug at time of implant. Pumps were implanted through a small incision in the skin between the scapulae into a subcutaneous pocket made by blunt dissection. The skin incision was then closed with sutures and wound glue.

Tamoxifen treatment: HSA-MCM Numbf/f/NumbLf/f mice were injected intraperitoneally with 0.2 mL of tamoxifen solution (10 mg/mL in peanut oil/5% ethanol) or vehicle for five consecutive days and weekly thereafter beginning on Day 10 after the first injection until they were sacrificed at 7, 14, 21, 28 and 35 days after the first injection. These mice are referred to as Numb/NumbL cKO and vehicle-treated controls, respectively. cKO, Numbf/f/NumbLf/f mice treated with tamoxifen or vehicle were used as tamoxifen-treated or vehicle-treated genotype controls, respectively. The younger mice were 4 to 6 months of age and older mice 12 to 14 months of age at the time of the first injection of vehicle or tamoxifen.

### Tissue Collection and Euthanasia

At times indicated in the Figure legends, while under isoflurane anesthesia induction and maintained as above, muscles (gastrocnemius (Gas), soleus (Sol), plantaris (Pl), and extensor digitorum longus (EDL) from the left hindlimb were removed by careful dissection, weighed and frozen by submersion in ice-cold isopentane (Sigma, 78-78-4) cooled with liquid nitrogen. Animals were euthanized by exsanguination.

### Experiment 1

77 males were used for these experiments because they had been used in our prior experiments evaluating effects of nandrolone on muscle mass after denervation in rats 8, 25, 26 and because the androgens used are likely to be virilizing in females. Mice underwent denervation or sham denervation with implantation of Alzet mini osmotic pumps (Alzet, Model 1004). Pumps infused either nandrolone (0.75 mg/kg/week in 5% ethanol in propylene glycol) or 5% ethanol propylene glycol. Tissues were collected at 7, 14 and 21 days thereafter.

### Experiment 2

Two cohorts of mice were studied. In one, 58 male mice were used for a prevention design. Alzet mini osmotic pumps (Alzet, Model 1002) were implanted at the time of denervation and mice were euthanized for tissue collection 7 days later. In the second, 29 male mice were used for a treatment design in which implantation of Alzet model 1002 pumps was done at post-denervation day 29 under anesthesia with isoflurane as above and mice were euthanized 7 days later at day 35 post-nerve transection. Each pump infused 0.75mg/kg/week nandrolone and 2.8 mg/kg/week testosterone in 5% ethanol in propylene glycol or 5% ethanol in propylene glycol alone. Testosterone was included because it was also used in our original studies in rats in which a slight protective effect of nandrolone was shown when administered beginning at day 29 after denervation 8.

### Experiment 3

Male HSA-MCM/Numb^(f/f)^/NumbL^(f/f)^ at ages from 12 to 20 weeks at time of induction 60 mice were used for these studies. Induction of Numb/NumbL knockdown was done by intraperitoneal injection of 0.2 ml of vehicle (Veh, 5% ethanol in peanut oil) or 2 mg of tamoxifen in Vehicle (TAM) daily for 5 days with booster injections done weekly thereafter until the time of euthanasia. Mice underwent either a Left (L) sciatic nerve transection or L sham transection at day 14 after inducing Numb/NumbL knockdown. Mice underwent tissue collection and euthanasia at 7, 14 or 28 days later as described above.

### Protein extraction, enrichment of nuclear protein and western blotting

Gastrocnemius muscle (25 mg) was homogenized in 500 μl of lysate buffer [150 mM sodium chloride, 3.2 mM Na2PO4, 0.8 m K_2_PO_4_(pH 7.4),1% NP40, 0.5% sodium deoxycholate, 0.5% sodium dodecyl sulfate] using a Polytron. Homogenates were cleared by centrifugation in a microcentrifuge at 14,000 rpm for 5 min. Protein content was assayed using a Bio-Rad protein assay kit (Hercules, CA) with bovine serum albumin as a standard. Western blotting was performed as previously described 27. β-Tubulin was used as the internal control for Western blot analyses of total proteins. Immunostaining was visualized by enhanced chemiluminescence. Scanning densitometry of digitized images was performed using Image quant TL (GE Life Sciences, Piscataway, NJ). Intensities of bands were normalized relative to β-tubulin as indicated then expressed as fold-change relative to the Sham-denervated.

### RT-qPCR

Total RNA was exacted using RNAeasy mini kits (QIAGEN). 1 μg of total RNA was used to synthesize cDNA using High-Capacity RNA‐to‐cDNA kit (Applied Biosystems). Relative expression of mRNA was determined by *q*Rt‐PCR, as described previously (PMID:31250564) using a thermocycler (model ViiA7, Applied Biosystems). Specific primers and *Tag*Man Universal Master Mixer were purchased from Applied Biosystems. *Tag*Man Reaction Mixes contained 3 μL cDNA (10 ng/μL). For each sample, the determinations were performed in triplicate, and the means for the crossing points of triplicates were used in subsequent calculations. Relative mRNA levels were expressed as fold‐change using the 2^-ΔΔCt^ method. Data were normalized relative to 18s RNA.

### Statistics

Data are expressed as mean values ± standard error. Significance of differences between means was determined using one-way ANOVA with a Tukey's test post-hoc. P values of less than 0.05 were considered significant.

## Results

### Experiment 1

**Figure 1:**
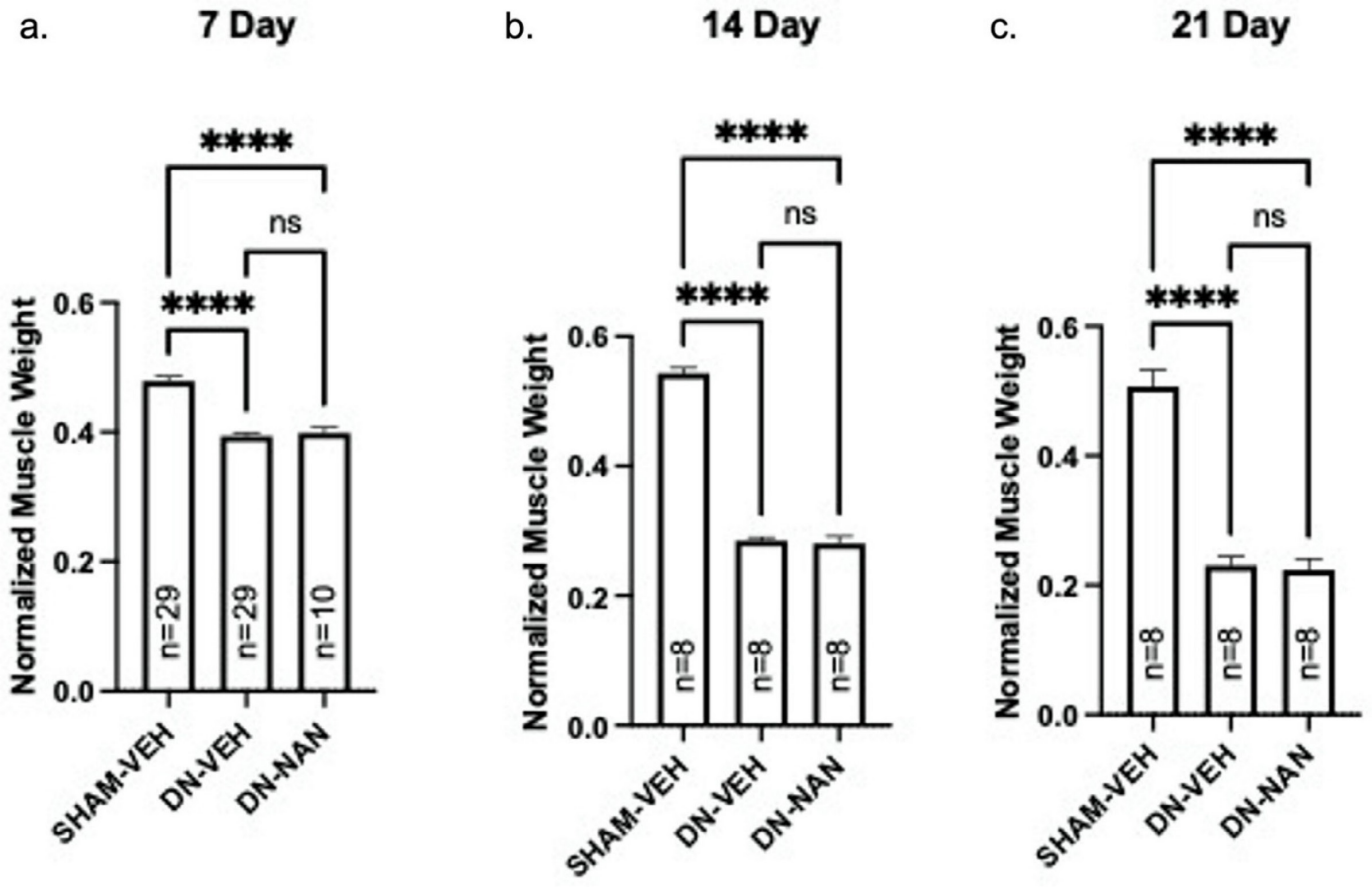
** Effect of nandrolone on muscle atrophy over time after sciatic nerve transection.** Continuous infusion of nandrolone (0.75 mg/kg/week) on muscle atrophy over time was tested in male C57B6J mice. Following denervation, gastrocnemius muscle weights were significantly reduced at 7 days (18%, p < 0.0001, 14 days (48%, p < 0.0001) and 21 days (55%, p < 0.0001) mice were euthanized at 7, 14 or 21 days after nerve transection followed by removal of gastrocnemius muscles which were weighed. Gastrocnemius weights are expressed mean ± SEM after normalization using pre-operative body weight. ****, p < 0.0001; ANOVA with a Tukey's test post-hoc.

Effects of a continuous infusion of nandrolone (0.75 mg/kg/week) on muscle atrophy over time was tested in male C57B6J mice. Following denervation, gastrocnemius muscle weights were significantly reduced at 7 days (18%, p < 0.0001, 14 days (48%, p < 0.0001) and 21 days (55%, p < 0.0001) as shown in Fig. 1a-c. Weights of denervated muscle from nandrolone-treated mice were not different from those from denervated gastrocnemius from vehicle-treated at any of these time points (Fig. 1a-c). A similar pattern of atrophy was observed for EDL and soleus muscles for which, again, no protection against denervation atrophy was provided by nandrolone (Supplementary Fig. 1). Thus, nandrolone alone did not afford any protection against denervation atrophy in C57B6J mice.

### Experiment 2

**Figure 2:**
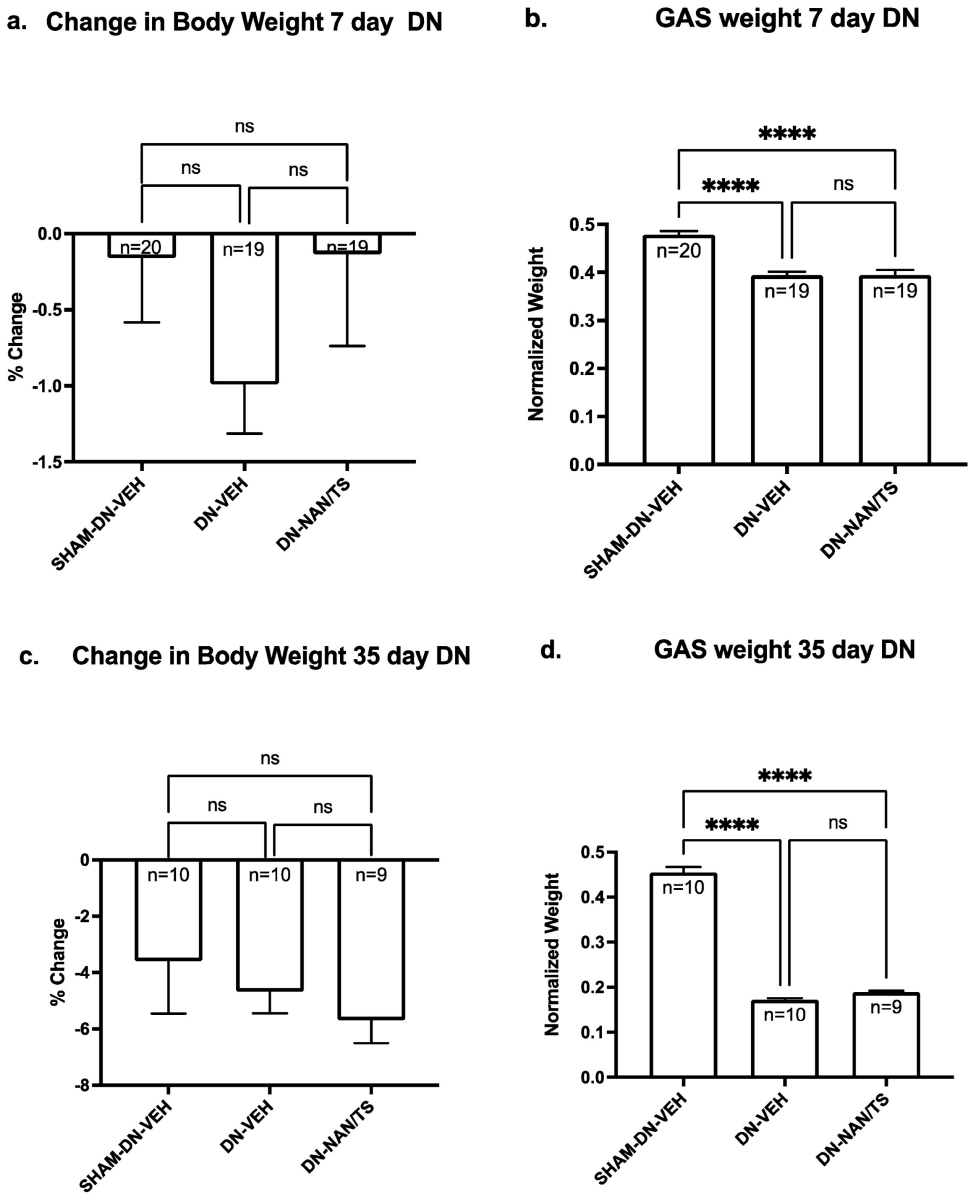
** Effect of nandrolone on muscle atrophy over time after sciatic nerve transection.** 7d (a) and 35 d (c) demonstrates change in body weight - Mice were euthanized at 7 and 35 days after nerve transection followed by removal of gastrocnemius muscles which were weighed. Gastrocnemius weights 7 d (b) and 35 d (d) are expressed mean ± SEM after normalization using pre-operative body weight. ****, p < 0.00001; ANOVA with a Tukey's test post-hoc.

We then determined using male C57B6J mice if seven days of nandrolone treatment combined with a low dose of testosterone reduced muscle atrophy when initiated at the time denervation surgery or at day 29. This experimental design was chosen because in our prior studies with rats we observed using a treatment approach that combined nandrolone (0.75 mg/kg/week) with testosterone slowed denervation atrophy when started at day 29 after nerve transection 8. When comparing the extent of atrophy between gastrocnemius (Fig. 2) versus soleus and EDL muscles (Supplementary Fig. 2), the most severe atrophy was observed in gastrocnemius. Consistent our findings for with experiment 1, at seven days after denervation, gastrocnemius muscle mass was approximately 18% lower than mass of sham-denervated gastrocnemius (Fig. 1, p<0.0001) and was not altered by a seven-day treatment with nandrolone plus testosterone (Fig. 2a, p = 0.9985). At thirty-five days after denervation, gastrocnemius muscle mass was reduced by 62% when compared to sham-denervated gastrocnemius muscle (Fig. 2d, p < 0.0001) and a seven-day treatment with nandrolone/testosterone (Nan/TS) slightly (9.9%) though non-significantly increased the gastrocnemius muscle mass (Fig. 2b, p = 0.263). Nan/TS did not significantly alter weights of denervated EDL or soleus at either 7 or 35 days after denervation (Supplementary Fig. 2a-d).

**Figure 3:**
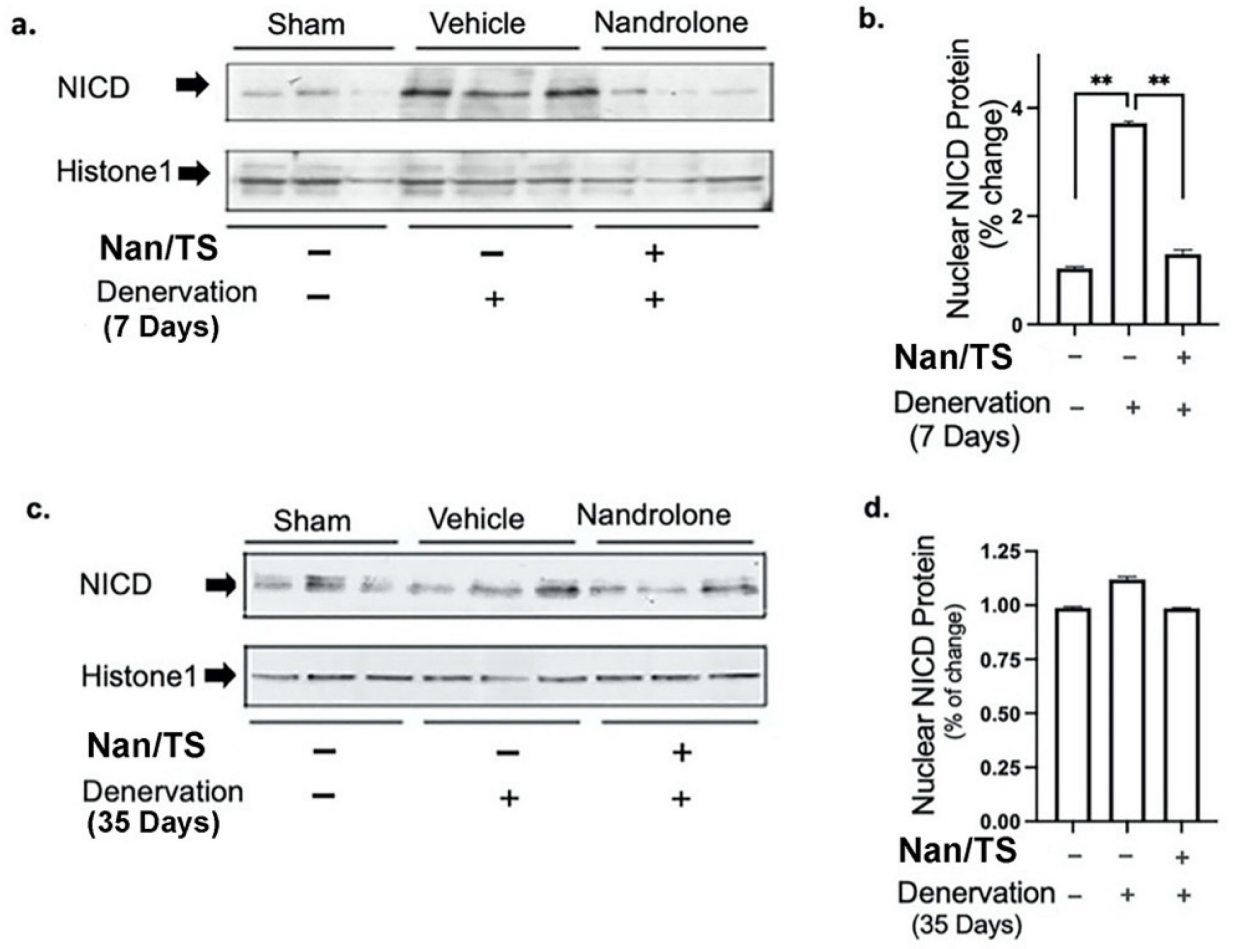
** Effect of nandrolone on denervation-induced nuclear Notch Intracellular Domain (NICD) protein expression.** Nuclear protein was isolated from mouse gastrocnemius muscles harvested from animals used in experiment 2 at 7d (a, b) denervation 35d (c, d). Samples were subjected to Western blot analysis using an antibody against NICD, the blots were then stripped and re-probed with antibody against histone H1. Band intensities in *a and c* were quantified by scanning densitometry and normalized to histone H1. Data shown in *b and d* are means ± SEM for 3 animals per group. ** *p* < 0.01; ANOVA with a Tukey's test post-hoc.

**Figure 4:**
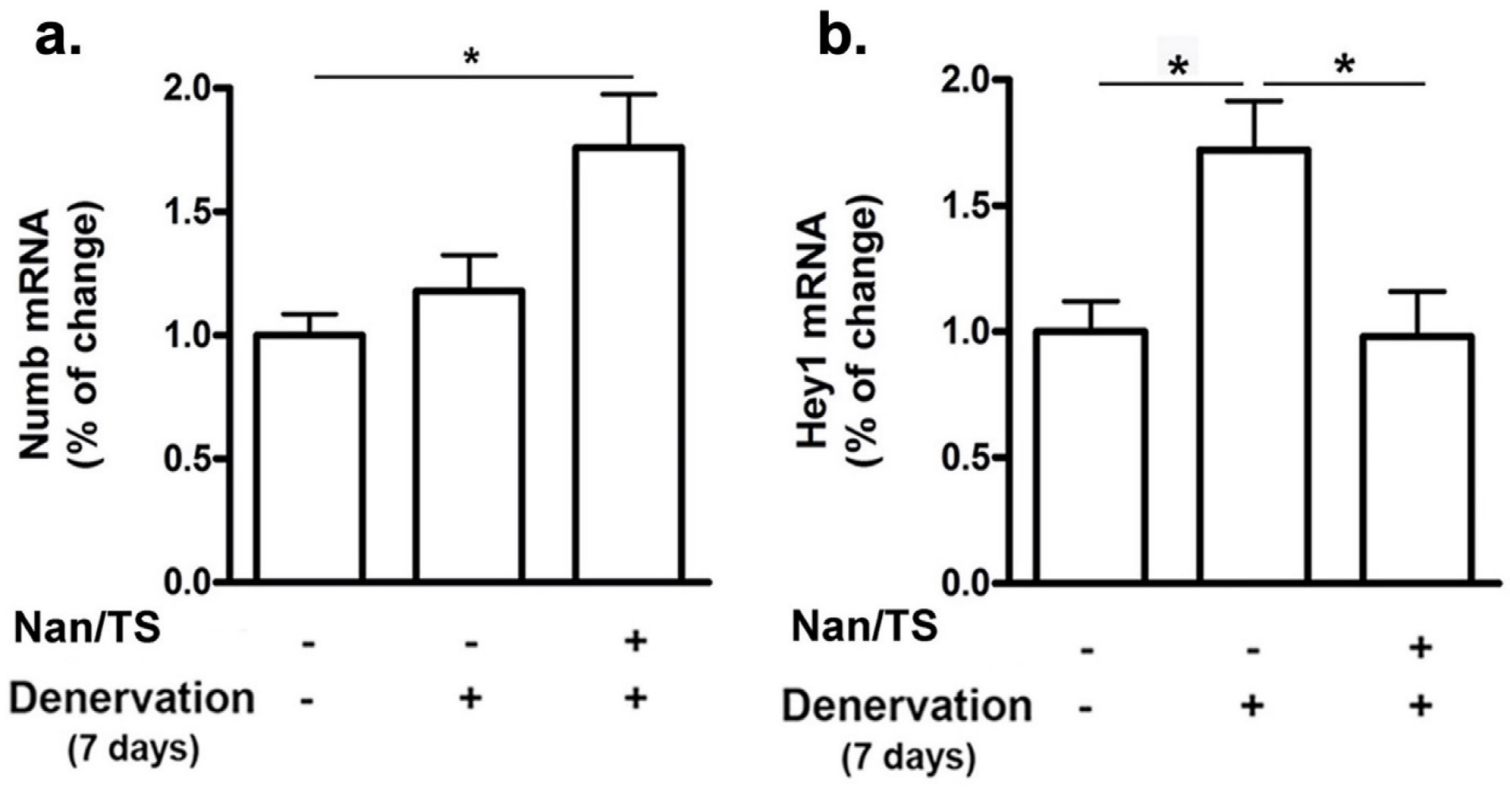
** Effect of Nandrolone/testosterone on mRNA expression of Numb and Hey1 after denervation.** Total RNA was isolated from mouse gastrocnemius muscles harvested from animals used in experiment 2 after 7 d denervation and subjected to Rt-PCR analysis. *(a)* Numb mRNA; *(b)* Hey1 mRNA. Data shown are means ± SEM for 4 animals per group. * *p* < 0.05; ANOVA with a Tukey's test post-hoc.

**Figure 5:**
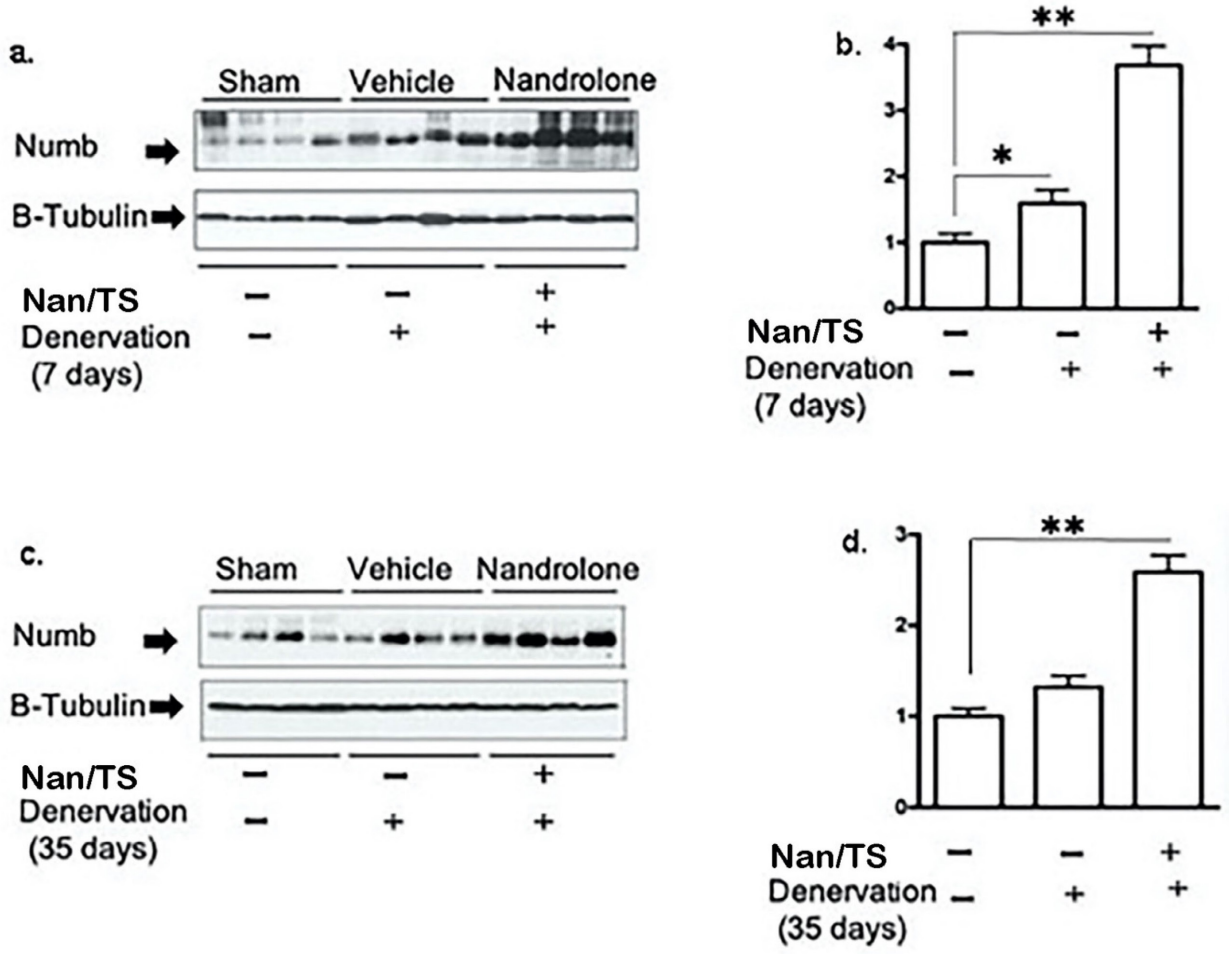
** Effect of nandrolone/Testosterone on denervation-induced Numb protein expression.** Total protein was isolated from mouse gastrocnemius muscles harvested from animals used in experiment 2 after 7 d (a, b, cohort 1) and 35 d (c, d, cohort 2) denervation. Samples were subjected to Western blot analysis using an antibody against Numb; the blots were then stripped and re-probed with antibody against β-tubulin. Band intensities in *a and c* were quantified by scanning densitometry and normalized to β-tubulin. Data shown in *b and d* are means + SEM for 4 animals per group. * *p* < 0.05, and ** *p* < 0.01; ANOVA with a Tukey's test post-hoc.

We next examined effects of Nan/TS on Notch signaling in gastrocnemius muscle from these same mice at 7 and 35 days after transection. Notch intracellular domain (NICD) levels were significantly elevated in nuclei isolated from frozen denervated gastrocnemius muscle collected from vehicle-treated mice at 7 days after nerve transection (Fig. 2) as compared to sham transected animals (Fig. 1a-b) but were not different when comparing sham versus denervated muscle from vehicle-treated mice at 35 days (Fig. 3c-d). At seven days after nerve transection, nuclear NICD levels were significantly lower in denervated gastrocnemius from Nan/TS treated mice as compared to denervated muscle from vehicle-treated mice (Fig. 3a-b). At thirty-five days after denervation, NICD levels were not significantly different when comparing denervated muscle from Nan/TS treated and vehicle-treated mice (Fig. 3c-d). Expression of Hey1, a downstream target of NICD, 8 was tested in gastrocnemius muscle at 7 days after denervation. As compared to sham-denervated muscle, Hey1 mRNA levels were increased by 75 % in denervated vehicle-treated mice (Fig. 4a) and were significantly reduced by treatment with Nan/TS (Fig 4a). Expression of Numb mRNA was significantly increased in denervated muscle from mice treated with Nan/TS when compared to denervated muscle from vehicle-treated mice at 7 days (Fig. 4b) but was not different when comparing denervated and sham-denervated muscle from vehicle-treated mice (Fig. 4b). Numb protein levels were not different when comparing muscle from denervated versus sham-denervated vehicle-treated mice at either 7 or 35 days after denervation (Fig. 5a-b). Nan/TS significantly increased Numb protein levels in denervated muscle as compared to denervated muscle from vehicle-treated mice at both 7 days after denervation by more than 3-fold and at 35 days after denervation by more than 2-fold (Fig. 5a-b).

### Experiment 3

**Figure 6:**
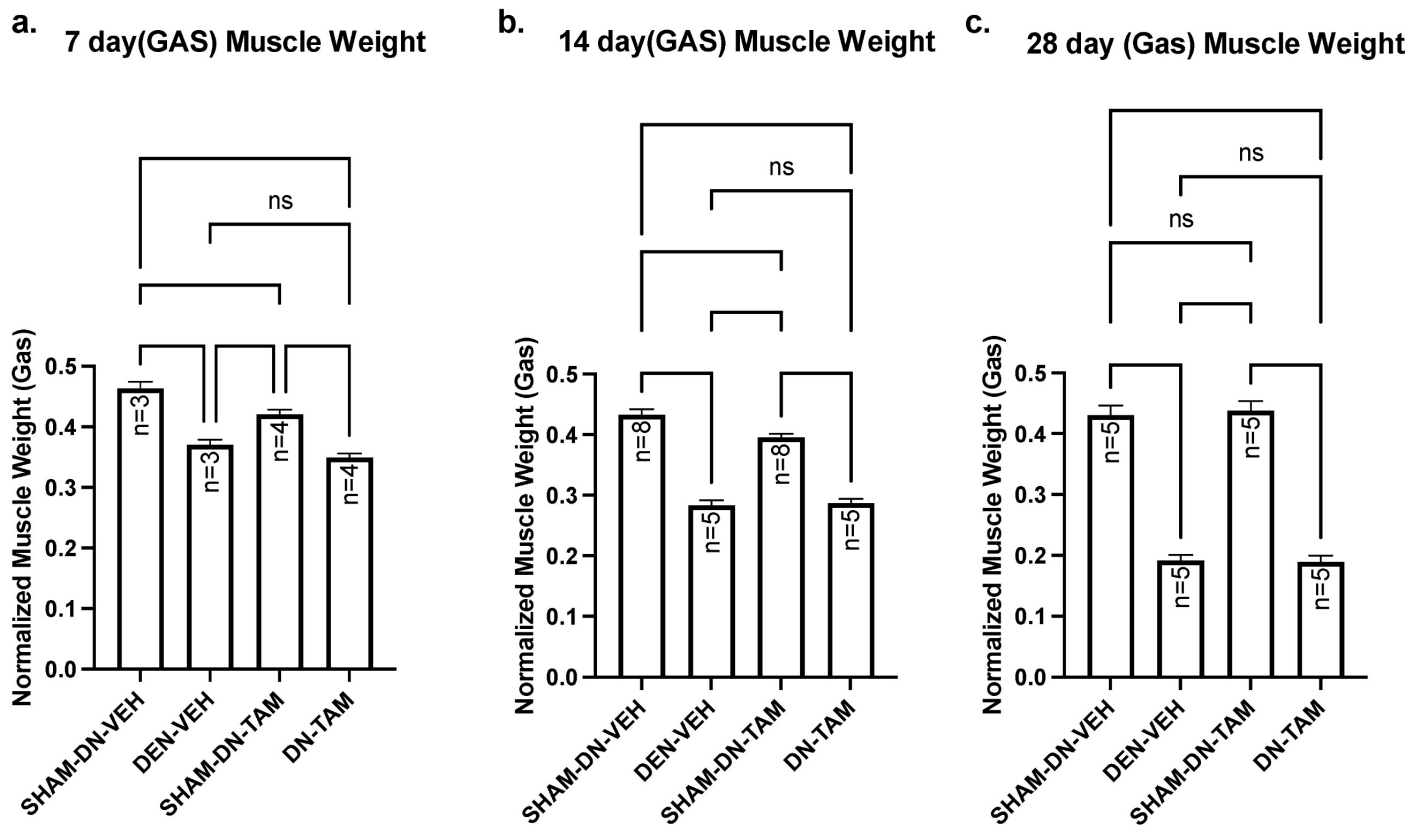
Muscle weight comparison of left denervated gastrocnemius with right sham-denervated control in tamoxifen treated versus vehicle treated. Data are mean ± SEM. **, *p* < 0.01; ***, *p* < 0.001; one-way ANOVA with a Tukey's test post-hoc.

The above findings indicate that nandrolone increases Numb expression in denervated gastrocnemius muscle from mice and this is associated with reduced levels of NICD and of Hey1 mRNA expression. These changes, however, do not slow the course of denervation atrophy indicating that raising Numb levels does not alter the trajectory of denervation atrophy and suggesting that elevated NICD/Hey1 do not drive such atrophy. We recently demonstrated that Numb is present within myofibers and that a conditional inducible deletion of Numb in myofibers results in reduced muscle force generation, disorganization of sarcomeres and defects in mitochondrial size and function 24. We therefore asked whether physiological levels of Numb within myofibers would protect against denervation atrophy. HSA-MCM/Numb^(f/f)^/NumbL^(f/f)^ mice allow the conditional, inducible knockdown of Numb in skeletal muscle myofibers while not affecting muscle progenitor cells 24. HSA-MCM/Numb^(f/f)^/NumbL^(f/f)^ mice were randomized to either induction of Numb knockouts by tamoxifen treatment or treatment with vehicle. At fourteen days after the start of tamoxifen or vehicle treatment, mice underwent a left sciatic nerve transection and right sham-transection in which the nerve was exposed but not manipulated and mice were euthanized 7, 14 or 28 days later. When comparing the left denervated gastrocnemius muscle weights with those for the right sham-denervated control in the vehicle-treated cohorts, weights of the denervated muscle were significantly reduced by amounts similar to that previously observed for C57B6J mice. Specifically, muscle weights of denervated gastrocnemius isolated from vehicle-treated HSA-MCM/Numb^(f/f)^/NumbL^(f/f)^ mice were reduced by approximately 21% at 7 days (Fig. 6a), 35% at 14 days (Fig. 6b) and 56% at 28 days (Fig. 6c). For tamoxifen-treated HSA-MCM/Numb^(f/f)^/NumbL^(f/f)^ mice reduced about 20% between sham and denervated muscle at 7 days (Fig. 6a); by 28% and 57% at 14 and 28 days, respectively (Fig. 6a-c). When comparing the right sham-denervated gastrocnemius muscle weights between the vehicle-treated and tamoxifen-treated groups, muscle weights were slightly reduced in the tamoxifen-treated mice at 7 and 14 days after surgery but were not different at 28 days after surgery (Fig. 6a-c). When comparing gastrocnemius muscle weights between denervated vehicle and denervated tamoxifen-treated groups, no differences in weights were observed at any time of the points evaluated (Fig. 6a-c). Total protein was isolated from gastrocnemius muscle and plantaris muscle. HSA‐MCMNumb^f/f^/NumbL^f/f^ mice were treated with vehicle or tamoxifen and sacrificed 21 days post‐induction, which corresponds 7 days after denervation. (*a*) Western blot analysis showed reduced levels of Numb protein in gastrocnemius muscle and plantaris muscle (Supplementary Fig. 3).

## Discussion

The above findings support several conclusions. In mice, as was true in rats, denervation induces activation of Notch 1 signaling which was reflected in increased nuclear NICD levels and Hey1 mRNA expression. Nandrolone plus testosterone increased Numb expression in denervated muscle at 7 and 35 days and blocked the increases in nuclear NICD and Hey1 mRNA. In contrast to our prior findings in rats, nandrolone alone or combined with testosterone did not significantly slow denervation atrophy. Conditional, inducible knockout of Numb and NumbL did not alter the rate of denervation atrophy. Taken together, the findings indicate that denervation atrophy is uncoupled from Numb expression levels, NICD levels or Hey1 expression. Thus, the data support the conclusion that neither Numb expression levels nor Notch 1 activation contributes significantly to denervation atrophy.

There are several caveats to consider when interpreting results of the above experiments. The first is that the cell type(s) in which changes in nuclear NICD occurred were not defined; immunofluorescence studies and confocal microscopy approaches were not successful in detecting nuclear NICD in these tissues. The second is that a limitation of our system for knocking down Numb expression in myofibers uses doses of tamoxifen that slightly reduce muscle weights which could mask a small effect of the Numb knockdown on rates of muscle atrophy. Despite this limitation, the dramatic atrophy observed in vehicle and tamoxifen treated mice at 28 days does suggest that the knockdown of Numb in myofibers did not appreciably alter the trajectory of denervation atrophy.

The findings in mice largely recapitulated our prior observations in rats showing that denervation induced transient upregulation of Notch 1/Hey1 signaling that was prevented by nandrolone plus testosterone in association with increased Numb expression. In our prior studies in rats, we observed a slight though significant slowing of denervation atrophy when nandrolone plus testosterone treatment was initiated at day 29 after nerve transection. While we found no significant difference in weights of denervated muscle between nandrolone-testosterone-treated mice and vehicle-treated mice at any time point, a trend toward a slight protection against atrophy was noted in mice for which treatment was started at 29 days. It is possible that with a larger group size this difference would become statistically significant.

In summary, using an approach whereby Numb expression levels were increased pharmacologically or reduced genetically, no effects of Numb levels on progression of denervation atrophy were observed. Furthermore, in pharmacologic studies, Numb expression, NICD levels and Hey1 expression were coupled, but were not related to progression of denervation atrophy. Collectively, the data do not support a role for Notch 1/Hey1 signaling in driving denervation atrophy or indicate a role for Numb in protecting against denervation atrophy. Because aging reduces expression of Numb 28, one question raised by our data is whether the progression of muscle fiber atrophy resulting from denervation caused by the aging process is altered by the reduced expression of Numb in muscle of old organisms. Further investigation is needed to answer this question.

## Supplementary Material

Supplementary figures.

## References

[1] Carlson BM (2014). The Biology of Long-Term Denervated Skeletal Muscle. Eur J Transl Myol.

[2] Bodine SC, Latres E, Baumhueter S, Lai VK, Nunez L, Clarke BA (2001). Identification of ubiquitin ligases required for skeletal muscle atrophy. Science.

[3] Bodine SC, Baehr LM (2014). Skeletal muscle atrophy and the E3 ubiquitin ligases MuRF1 and MAFbx/atrogin-1. Am J Physiol Endocrinol Metab.

[4] Jackman RW, Kandarian SC (2004). The molecular basis of skeletal muscle atrophy. Am J Physiol Cell Physiol.

[5] Cea LA, Cisterna BA, Puebla C, Frank M, Figueroa XF, Cardozo C (2013). De novo expression of connexin hemichannels in denervated fast skeletal muscles leads to atrophy. Proc Natl Acad Sci U S A.

[6] Cisterna BA, Vargas AA, Puebla C, Saez JC (2016). Connexin hemichannels explain the ionic imbalance and lead to atrophy in denervated skeletal muscles. Biochim Biophys Acta.

[7] Cisterna BA, Vargas AA, Puebla C, Fernandez P, Escamilla R, Lagos CF (2020). Active acetylcholine receptors prevent the atrophy of skeletal muscles and favor reinnervation. Nat Commun.

[8] Zhao J, Zhang Y, Zhao W, Wu Y, Pan J, Bauman WA (2008). Effects of nandrolone on denervation atrophy depend upon time after nerve transection. Muscle Nerve.

[9] Liu XH, Yao S, Qiao RF, Levine AC, Kirschenbaum A, Pan J (2011). Nandrolone reduces activation of Notch signaling in denervated muscle associated with increased Numb expression. Biochem Biophys Res Commun.

[10] Camerino GM, Desaphy JF, De Bellis M, Capogrosso RF, Cozzoli A, Dinardo MM (2015). Effects of Nandrolone in the Counteraction of Skeletal Muscle Atrophy in a Mouse Model of Muscle Disuse: Molecular Biology and Functional Evaluation. PLoS One.

[11] Bray SJ (2016). Notch signalling in context. Nature Reviews Molecular Cell Biology.

[12] Brack AS, Conboy IM, Conboy MJ, Shen J, Rando TA (2008). A temporal switch from notch to Wnt signaling in muscle stem cells is necessary for normal adult myogenesis. Cell Stem Cell.

[13] Conboy IM, Rando TA (2002). The regulation of Notch signaling controls satellite cell activation and cell fate determination in postnatal myogenesis. Dev Cell.

[14] Bi P, Yue F, Sato Y, Wirbisky S, Liu W, Shan T (2016). Stage-specific effects of Notch activation during skeletal myogenesis. Elife.

[15] Mu X, Agarwal R, March D, Rothenberg A, Voigt C, Tebbets J (2016). Notch Signaling Mediates Skeletal Muscle Atrophy in Cancer Cachexia Caused by Osteosarcoma. Sarcoma.

[16] Gulino A, Di Marcotullio L, Screpanti I (2010). The multiple functions of Numb. Exp Cell Res.

[17] Yang J, Bucker S, Jungblut B, Bottger T, Cinnamon Y, Tchorz J (2012). Inhibition of Notch2 by Numb/Numblike controls myocardial compaction in the heart. Cardiovasc Res.

[18] Di Marcotullio L, Ferretti E, Greco A, De Smaele E, Po A, Sico MA (2006). Numb is a suppressor of Hedgehog signalling and targets Gli1 for Itch-dependent ubiquitination. Nat Cell Biol.

[19] Pece S, Confalonieri S, P RR, Di Fiore PP (2011). NUMB-ing down cancer by more than just a NOTCH. Biochim Biophys Acta.

[20] Flores AN, McDermott N, Meunier A, Marignol L (2014). NUMB inhibition of NOTCH signalling as a therapeutic target in prostate cancer. Nat Rev Urol.

[21] Katoh M (2006). NUMB is a break of WNT-Notch signaling cycle. Int J Mol Med.

[22] Liu XH, Wu Y, Yao S, Levine AC, Kirschenbaum A, Collier L (2013). Androgens up-regulate transcription of the Notch inhibitor Numb in C2C12 myoblasts via Wnt/beta-catenin signaling to T cell factor elements in the Numb promoter. J Biol Chem.

[23] George RM, Biressi S, Beres BJ, Rogers E, Mulia AK, Allen RE (2013). Numb-deficient satellite cells have regeneration and proliferation defects. Proc Natl Acad Sci U S A.

[24] De Gasperi R, Mo C, Azulai D, Wang Z, Harlow LM, Du Y (2022). Numb is required for optimal contraction of skeletal muscle. J Cachexia Sarcopenia Muscle.

[25] Liu XH, Yao S, Qiao RF, Levine AC, Kirschenbaum A, Pan J (2011). Nandrolone reduces activation of Notch signaling in denervated muscle associated with increased Numb expression. Biochem Biophys Res Commun.

[26] Qin W, Pan J, Bauman WA, Cardozo CP (2010). Differential alterations in gene expression profiles contribute to time-dependent effects of nandrolone to prevent denervation atrophy. BMC Genomics.

[27] Cardozo CP, Qin W, Peng Y, Liu X, Wu Y, Pan J (2010). Nandrolone slows hindlimb bone loss in a rat model of bone loss due to denervation. Ann N Y Acad Sci.

[28] Balistreri CR, Madonna R, Melino G, Caruso C (2016). The emerging role of Notch pathway in ageing: Focus on the related mechanisms in age-related diseases. Ageing Res Rev.

