# Supplementary material for: Denervation Atrophy of Skeletal Muscle is Not Influenced by Numb Levels in Mice
Source: Int J Med Sci. 2023 Jan 31;20(3):376–84. doi: 10.7150/ijms.77603 (PMC9969502; doi:10.7150/ijms.77603)
Supplement: Supplementary file 1 — Supplementary figures. [file ijmsv20p0376s1.pdf]

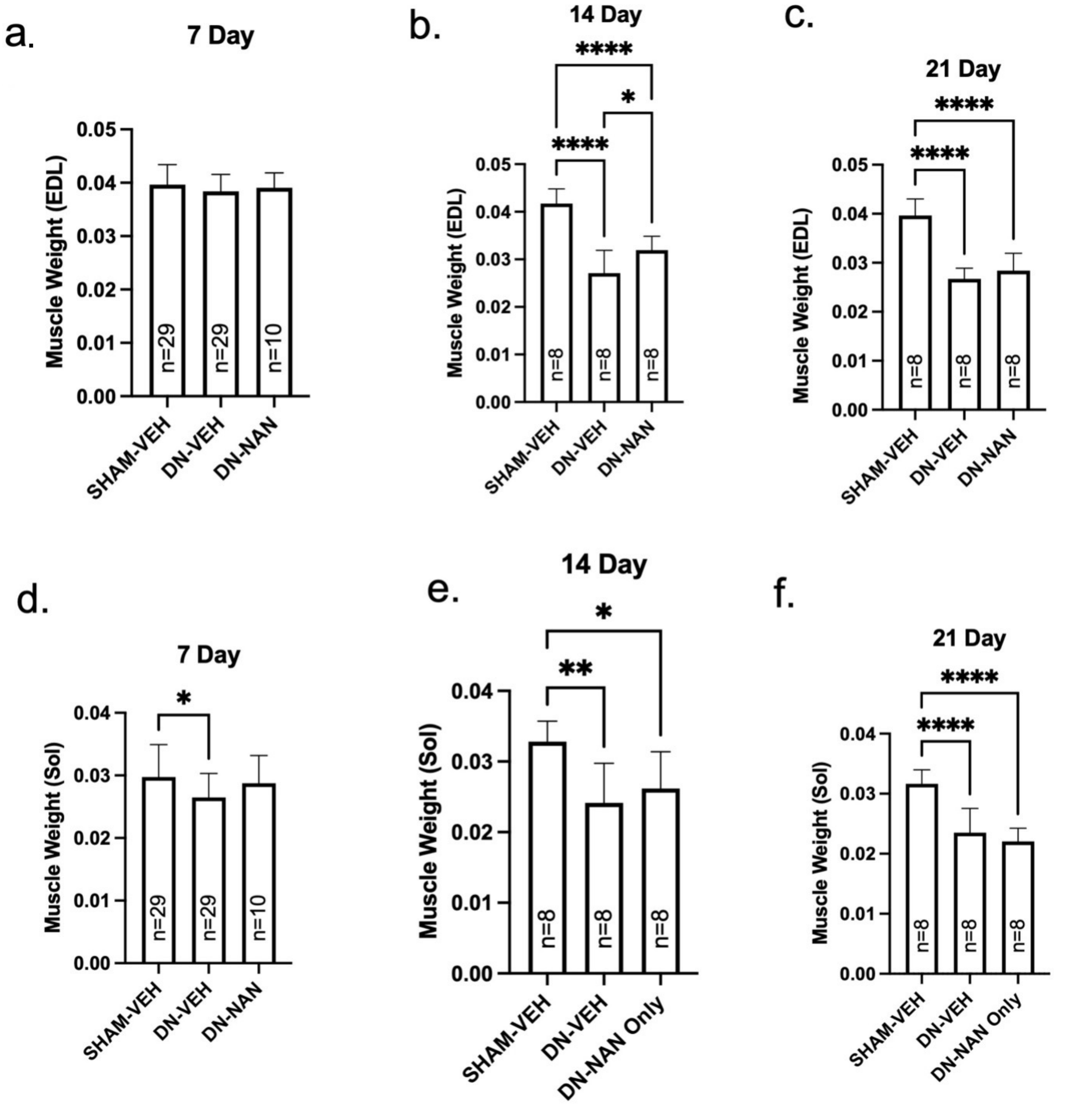

Suppl. Fig 1: This figure shows effect of nandrolone on muscle atrophy over time after sciatic nerve transection. Continuous infusion of nandrolone (0.75 mg/kg/week) on muscle atrophy over time was tested in male C57B6J mice. Following denervation, EDL and Sol muscle weights shows the same pattern of denervation atrophy as was Figure1 for GAS muscle at 7, 14 or 21 days after nerve transection. EDL and Sol weights are expressed mean ± SEM after normalization using pre-operative body weights. *$p<0.05$, **$p<0.001$, ****, $p < 0.0001$; ANOVA with a Tukey's test post-hoc.

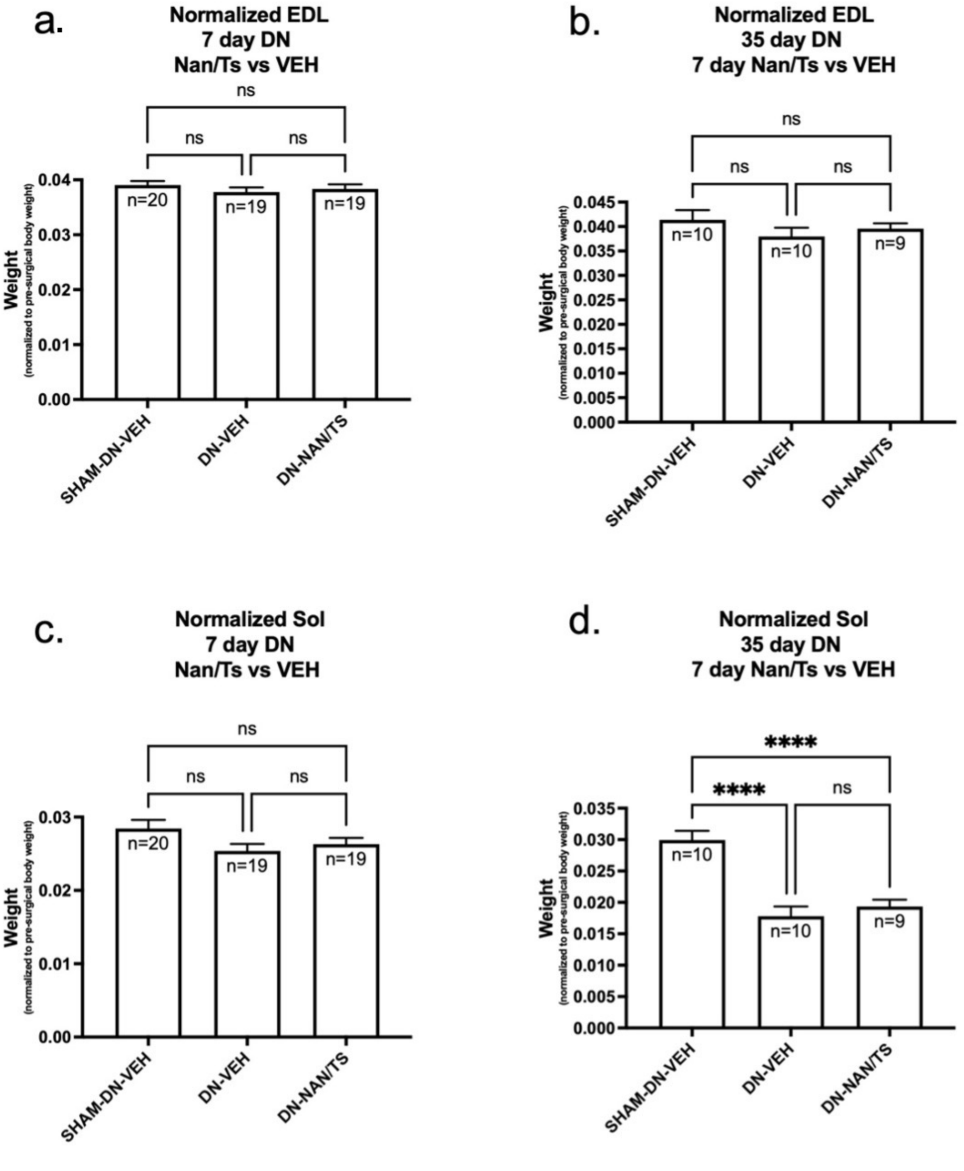

Suppl. Figure 2: *Effect of nandrolone on muscle atrophy over time after sciatic nerve transection. 7d (a,c) and 35 d (b,d) demonstrates change in muscle weight of EDL and Soleus (Sol)* – Mice were euthanized at 7 and 35 days after nerve transection followed by removal of EDL and Sol muscles which were weighed. Nandrolone plus testosterone did not significantly alter weights of denervated EDL or soleus at either 7 or 35 days after denervation. EDL and Sol weights at 7 d (a) and 35 d (b) are expressed mean ± SEM after normalization using pre-operative body weight. ****, $p < 0.00001$; ANOVA with a Tukey's test post-hoc.

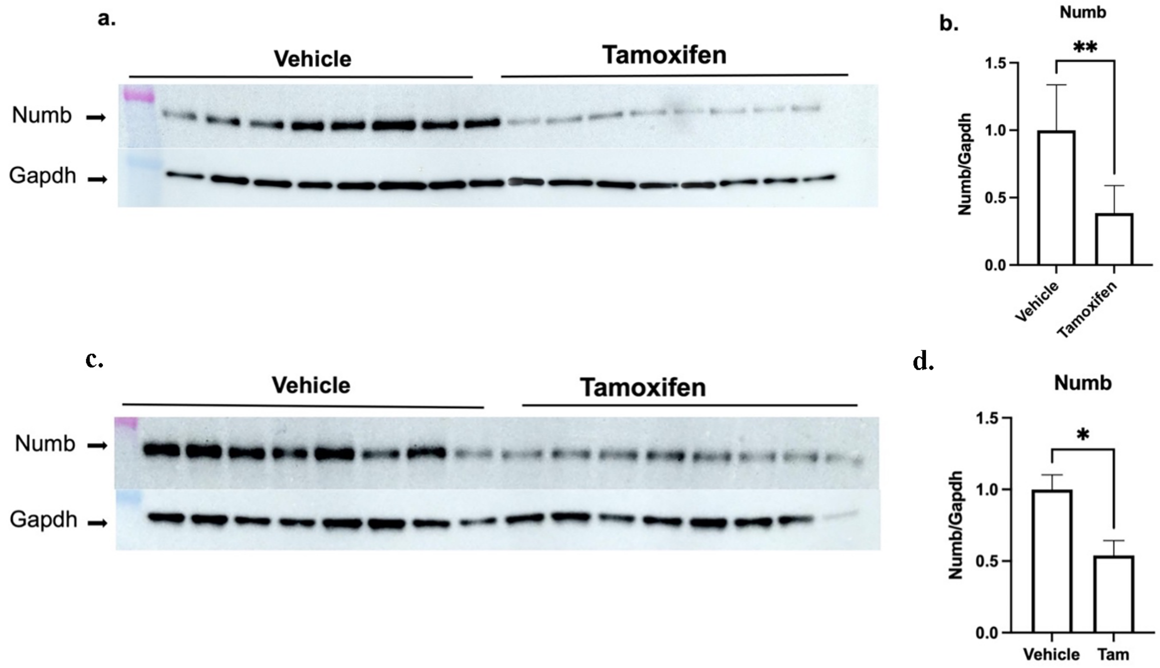

Suppl. Figure 3: Numb levels in denervated muscle. Total protein was isolated from gastrocnemius and plantaris muscle. HSA-MCM $Numb^{f/f}$/$NumbL^{f/f}$ mice were treated with vehicle or tamoxifen and sacrificed 21 days post-induction, which corresponds 7 days after denervation. Western blot analysis showing reduced levels of Numb protein shown in (a) gastrocnemius muscle and (c) plantaris muscle. Numb levels normalized with Gapdh ($N = 8$ per group). Quantitation of blots shown in (b) gastrocnemius and (d) plantaris muscles. Statistical analysis was performed two-tailed unpaired *t*-tests (b). $^{**}P < 0.01$, $^{*}P < 0.05$.

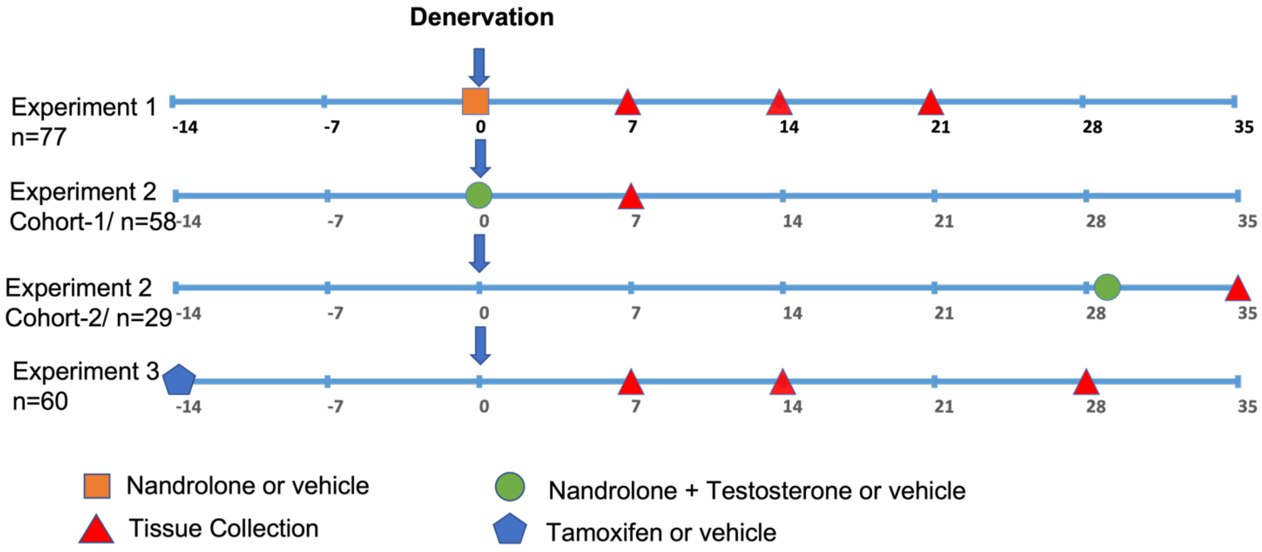

Suppl. Figure 4: Figure shows timeline (Day -14 to Day 35) of the experiments and number of animals used for each experiment.
